# Bridging thrombolysis with tenecteplase versus endovascular thrombectomy alone for large-vessel anterior circulation stroke: a target trial emulation analysis

**DOI:** 10.1136/jnnp-2024-335325

**Published:** 2025-01-22

**Authors:** Valerian L Altersberger, Johannes Kaesmacher, Leonid Churilov, Vignan Yogendrakumar, Jan Gralla, Daniel Strbian, David J Seiffge, Peter J Mitchell, Timothy J Kleinig, Bruce CV Campbell, Urs Fischer

**Affiliations:** 1 Department of Medicine and Neurology, Melbourne Brain Centre at The Royal Melbourne Hospital, University of Melbourne, Parkville, Victoria, Australia; 2 Department of Neurology and Stroke Center, University Hospital Basel and University of Basel, Basel, Switzerland; 3 University Institute of Diagnostic and Interventional Neuroradiology, University Hospital of Bern, Inselspital, University of Bern, Bern, Switzerland; 4 Division of Neurology, The Ottawa Hospital and Ottawa Research Institute, University of Ottawa, Ottawa, Ontario, Canada; 5 Department of Neurology, Helsinki University Hospital, and University of Helsinki, Helsinki, Finland; 6 Department of Neurology, University Hospital of Bern, University of Bern, Bern, Switzerland; 7 Department of Radiology, Royal Melbourne Hospital, Parkville, Victoria, Australia; 8 Department of Neurology, Royal Adelaide Hospital, Adelaide, South Australia, Australia

**Keywords:** STROKE, CEREBROVASCULAR DISEASE

## Abstract

**Background:**

Whether bridging thrombolysis with tenecteplase is beneficial compared with thrombectomy alone in patients who had a stroke with large-vessel occlusion remains unclear.

**Methods:**

This is a causal inference study of observational data from the trials SWIFT DIRECT and EXTEND-IA TNK Parts 1 and 2 applying target trial emulation. We compared patients receiving thrombectomy alone to patients receiving tenecteplase 0.25 mg/kg or 0.40 mg/kg before thrombectomy. The primary outcome was functional independence (modified Rankin Scale (mRS) of 0–2) at 90 days. Secondary outcomes included improvement over the full ordinal mRS scale, freedom of disability (mRS 0–1), mortality and occurrence of symptomatic intracranial haemorrhage. The average causal treatment effect was estimated via inverse probability of treatment weighting and G-Computation. We calculated standardised risk differences (SRDs) and adjusted (common) ORs (a(c)ORs).

**Results:**

Of 377 patients included in the target trial, 187 received thrombectomy alone and 190 tenecteplase before thrombectomy. Tenecteplase before thrombectomy did not increase the probability of patients achieving functional independence (SRD 0.04 (95% CI –0.06 to 0.13)) but resulted in a significant improvement in the mRS overall (acOR 1.56 (95% CI 1.07 to 2.23)) and in a higher probability of freedom from disability (SRD 0.10 (95% CI 0.01 to 0.20)). The probability for improvement of functional outcomes was further increased in patients treated within 140 min after onset (ordinal mRS acOR 1.63 (95% CI 1.04 to 2.56)). No significant differences in safety outcomes were observed between the two groups.

**Conclusion:**

Tenecteplase before thrombectomy compared with thrombectomy alone did not increase the probability of functional independence but resulted in significant improvement over the full mRS scale. This improvement was most evident in patients treated early.

**WHAT IS ALREADY KNOWN ON THIS TOPIC**

**WHAT THIS STUDY ADDS**

- This is the first causal inference study directly investigating the effect of tenecteplase as bridging thrombolytic before thrombectomy versus thrombectomy alone. Tenecteplase did not significantly increase the primary outcome of functional independence. However, in contrast to the randomised controlled trials and meta-analysis comparing thrombectomy alone versus alteplase as bridging thrombolytic before thrombectomy, bridging with tenecteplase was superior to thrombectomy alone with regard to freedom from disability and improvement in the full ordinal modified Rankin Scale at 90 days.

**HOW THIS STUDY MIGHT AFFECT RESEARCH, PRACTICE OR POLICY**

- Emulating a randomised controlled trial, our study provides evidence for the use of tenecteplase as a bridging thrombolytic, rather than thrombectomy alone, in patients with a large-vessel occlusion ischaemic stroke in the anterior circulation within the 4.5-hour time window. Validation of the results in randomised controlled trials is warranted.

## Introduction

 According to international stroke guidelines, standard care for patients presenting with a large-vessel occlusion ischaemic stroke is the administration of intravenous thrombolytic in eligible patients, alongside endovascular mechanical thrombectomy.^1^,^3^ As thrombolytics became well established before thrombectomy was proven to improve functional outcome, all participants in the positive thrombectomy trials received intravenous thrombolytic when eligible.^4^

An individual participant data meta-analysis of six recent randomised controlled trials, comparing thrombectomy alone versus intravenous thrombolytic before thrombectomy in patients with an anterior circulation large-vessel occlusion, neither found non-inferiority nor inferiority of thrombectomy alone.^5^,^11^ In these six trials, alteplase was used almost exclusively as the thrombolytic agent and only 25 patients (2.2%)—exclusively from one trial—were treated with tenecteplase, a genetically modified variant of alteplase with greater fibrin specificity and a longer half-life that allows bolus administration.^12^ Furthermore, the EXTEND-IA-TNK trial showed that tenecteplase (at a dose of 0.25 mg/kg) before thrombectomy was associated with a higher rate of reperfusion and better functional outcome when compared with alteplase (at a dose of 0.9 mg/kg).^13^

Randomised controlled data in patients treated with tenecteplase prior to thrombectomy versus thrombectomy alone are currently lacking. Therefore, we compared bridging thrombolytic with tenecteplase before thrombectomy to thrombectomy alone by emulating a target trial using individual patient data from three randomised controlled trials.

## Methods

### Study design and oversight

For this analysis, we emulated a hypothetical target trial according to previously published methods^14^,^16^ in which patients with a large-vessel occlusion ischaemic stroke in the anterior circulation were treated either with bridging thrombolytic with tenecteplase prior to thrombectomy or received thrombectomy alone (without prethrombectomy bridging thrombolytic). We used data from three randomised controlled trials: the Solitaire With the Intention for Thrombectomy Plus Intravenous t-PA vs DIRECT Solitaire StentRetriever Thrombectomy in Acute Anterior Circulation Stroke Trial (SWIFT DIRECT),^9^ the tenecteplase versus alteplase before endovascular thrombectomy trial (EXTEND-IA TNK)^13^ and the Determining the Optimal Dose of Tenecteplase Before Endovascular Therapy for Ischaemic Stroke Trial (EXTEND-IA TNK Part 2).^17^ The SWIFT DIRECT trial provided patients who received thrombectomy alone and the EXTEND-IA TNK trial and the EXTEND-IA TNK Part 2 trial provided patients who received tenecteplase before thrombectomy.

The collaborators of each trial agreed to participate in the study and the anonymised individual patient data were pooled. The characteristics of each trial are shown in online supplemental appendix eTable 1. This study followed the Strengthening the Reporting of Observational Studies in Epidemiology reporting guideline (online supplemental appendix emethods 1).

### Eligibility criteria

Target trial inclusion criteria were age ≥18 years, diagnosis of an acute ischaemic stroke with cerebral vascular occlusion on initial CT/MRI angiography of the intracranial internal carotid artery (ICA), the M1 segment of the middle cerebral artery (MCA) or both, eligibility for intravenous thrombolytic within 4.5 hours after stroke onset and direct presentation to a centre capable of performing thrombectomy. Additionally, we defined the following exclusion criteria: mild stroke severity at onset (defined as a baseline National Institutes of Health Stroke Scale (NIHSS) <5 points); intention not to perform thrombectomy; prestroke functional dependence (defined as a prestroke modified Rankin Scale (mRS) score >2); occlusions in multiple vascular territories on baseline CT/MRI angiography; pregnancy; any contraindication to intravenous thrombolytic according to international guidelines^1 2^; the presence of any intracranial haemorrhage; renal failure (defined as creatinine >3.0 mg/dL) or life expectancy <1 year. An overview of the inclusion and exclusion criteria of the target trial is presented in table 1.

**Table 1:** Inclusion and exclusion criteria for the target trial emulation

Target trial	
Inclusion	Exclusion
Patient enrolled in SWIFT-DIRECT and received mechanical thrombectomy alone Patient enrolled in EXTEND-IA TNK part 1 and 2 and received TNK 0.25 mg/kg or TNK 0.40 mg/kg before thrombectomy Direct admission to a centre capable of performing thrombectomy Intention to treat with thrombectomy Age ≥18 years Neurological deficit with a National Institutes of Health Stroke Scale score ≥5 points Prestroke independence (modified Rankin Scale 0–2) Occlusion in the anterior circulation: intracranial internal carotid artery and/or the M1 segment, of the middle cerebral artery Eligible for intravenous thrombolysis within 4.5 hours Eligible for thrombectomy Randomisation within 4.5 hours	Life expectancy <1 year Renal failure with creatinine >3.0 mg/dL Occlusion in multiple vascular territories Acute intracranial haemorrhage Any contraindication for intravenous thrombolysis Pregnancy

SWIFT-DIRECT, Solitaire With the Intention for Thrombectomy Plus Intravenous t-PA vs DIRECT Solitaire StentRetriever Thrombectomy in Acute Anterior Circulation Stroke Trial.

### Treatment

We defined treatment groups as (1) patients administered tenecteplase 0.25 mg/kg or 0.40 mg/kg before undergoing thrombectomy and (2) patients receiving thrombectomy alone without prior thrombolytic. In both treatment groups, thrombectomy was initiated as fast as possible with commercially available devices. In patients treated with tenecteplase before thrombectomy, the administration of tenecteplase was initiated as early as possible after randomisation.

### Outcomes, imaging and follow-up

Patients were followed up until death or 90 days after their respective stroke onset. The primary outcome was functional independence defined as a mRS score 0–2 at 90 days. Secondary outcomes included functional improvement across the full ordinal mRS (merging categories 5 and 6) at 90 days, freedom from disability (defined as a mRS score 0–1) at 90 days and early reperfusion prior to thrombectomy (defined as cross-sectional expanded Thrombolysis in Cerebral Infarction scale (eTICI) score of 2b–3, representing reperfusion of >50% of the originally involved vascular territory). In cross-sectional eTICI grading, the target downstream territory is defined based on the site of occlusion on pretreatment cross-sectional CT/MR angiography and the extent of reperfusion is defined based on the first run of subsequent digital subtraction angiography or non-invasive vascular imaging in patients with early neurological improvement.^18^ Images were graded by the respective trial core lab. Clinical outcomes were assessed by certified medical personnel during a clinical visit or a structured telephone interview who were blinded to treatment allocation.

Safety outcomes included mortality at 90 days and the occurrence of symptomatic intracranial haemorrhage (sICH). sICH was defined as any parenchymal haematoma type 1 (PH1), PH type 2 (PH2), remote intracranial haemorrhage, subarachnoid haemorrhage or intraventricular haemorrhage associated with a ≥4 point worsening on the NIHSS within 24 hours post-treatment.

To achieve optimal comparability between baseline Alberta Stroke Programme Early CT Score (ASPECTS) using CT versus MRI, we added one point if ASPECTS was evaluated on MRI and was <10, according to previously published literature.^19 20^

### Statistical analysis

Statistical analyses were performed with R V.4.3.3 (R Core Team, 2023). The prespecified statistical analysis plan is presented in online supplemental appendix eMethods 2. Considering the previously demonstrated improved functional outcome in patients receiving tenecteplase versus alteplase before thrombectomy,^13^ the primary research hypothesis was that tenecteplase 0.25 mg/kg or 0.40 mg/kg within 4.5 hours after stroke onset before thrombectomy would result in increased probability of functional independence (defined as mRS 0–2) at 90 days compared with receiving thrombectomy alone. We identified potential confounders with the use of directed acyclic graphs (DAGs) (online supplemental appendix eFigure 1). Cases with missing data for the primary outcome or confounding variables according to the DAGs were excluded. Subsequently, we estimated the average causal treatment effect of the treatment allocation (tenecteplase before thrombectomy vs thrombectomy alone) in primary and secondary outcomes by using standardisation to the population via:

1. Inverse probability of treatment weighting (IPTW) including previously identified covariates (ie, modelling the treatment).
2. G-Computation approach (ie, modelling the outcome).

To avoid non-random violation of the positivity assumptions, patients with a propensity score (estimated according to the IPTW model) outside the range of the propensity score of the other group (online supplemental appendix eFigure 2) were excluded from all analyses.

### Inverse probability of treatment weighting

For IPTW, we fitted a logistic regression model of the exposure tenecteplase before thrombectomy as a function of the following covariables (according to the DAGs): age, pretreatment NIHSS, ASPECTS on initial CT or MRI, location of the occluded vessel and time from onset to arterial puncture to generate propensity scores. We used the model’s estimated propensity scores to calculate stabilised inverse probability weights which were then used to weight each individual’s contribution to the logistic regression models of the respective primary and secondary outcomes of interest as dependent variables and exposure to tenecteplase before thrombectomy as independent variable, adjusted for the covariables listed above. Average causal effects were estimated as adjusted (common) ORs (a(c)OR) and standardised risk differences (SRDs) with respective 95% CIs.

### G-Computation

We fitted logistic regression models with the respective primary or secondary outcome as outcome variable, tenecteplase before thrombectomy as treatment variable and the potential confounding variables according to the DAGs as adjustment covariables. We then used these models to estimate the adjusted probabilities of achieving the respective primary and secondary outcomes under the scenarios when every patient was: (a) set to having received tenecteplase before thrombectomy and (b) set to not having received tenecteplase before thrombectomy. The average causal effect was estimated as the SRD between means of the respective adjusted estimated probabilities in the above two scenarios. Respective 95% CIs were obtained via delta method.

Secondary endpoints were analysed within the same model used for the primary outcome. The functional outcome across the full ordinal mRS scale (merging categories 5 and 6) was only analysed by IPTW calculating acORs as the analysis by G-Computation is not feasible for ordinal outcomes. The proportional odds assumption was tested using the Brant-Wald test.

### Sensitivity analysis

As tenecteplase 0.40 mg/kg resulted in worse functional outcome and increased rates of intracranial haemorrhage in the NOR-TEST 2A trial,^21^ we performed a prespecified subgroup sensitivity analysis for the primary and secondary outcomes only including patients treated with tenecteplase 0.25 mg/kg before thrombectomy and patients who received thrombectomy alone. A(c)ORs and SRDs with 95% CIs were estimated via IPTW for the primary and secondary outcomes.

### Subgroup analyses

#### Time to (expected) thrombolytic

To investigate a previously described time-dependent effect of bridging thrombolysis with alteplase on functional outcome in comparison to thrombectomy alone,^22^ we performed a prespecified subanalysis for the primary and secondary outcomes using (expected) onset-to-thrombolytic time as a dichotomous variable (within or after 140 min). In patients receiving thrombectomy alone, expected-onset-to-thrombolytic time was calculated by adding the mean time from randomisation to thrombolytic (derived from patients receiving bridging alteplase thrombolytic in the SWIFT DIRECT trial) to the time from onset to randomisation of each patient as done in previous research.^22^ In patients receiving tenecteplase before thrombectomy the actual onset-to-thrombolytic time was used. SRDs and a(c)ORs with 95% CIs were estimated via IPTW for the primary and secondary outcomes. Additionally, interaction between tenecteplase before thrombectomy and time to (expected) thrombolytic ≤140 min was investigated by introducing the relevant multiplicative term into the respective models. As (expected)-onset-to-thrombolytic time highly correlated with time from onset to arterial puncture, the latter was not used for calculating weights or adjusting the model. Post hoc, we also calculated a(c)ORs with 95% CIs for the primary and secondary outcomes using (expected) onset-to-thrombolytic time as a dichotomous variable with thresholds of 120 and 180 min.

#### Time from tenecteplase to thrombectomy

Prior studies have varied in whether shorter or longer time from thrombolytic to thrombectomy (dwell time) influences the benefit of bridging thrombolytic.^23 24^ Longer dwell time is associated with greater prethrombectomy reperfusion but some studies suggested greater benefit when the alteplase infusion was still running during thrombectomy.^25 26^ We, therefore, analysed the primary and secondary outcomes in subgroups defined by the time from tenecteplase to arterial puncture for thrombectomy (as a dichotomous variable within or after 30 min) in patients receiving bridging tenecteplase before thrombectomy. aOR or acOR and SRDs with 95% CIs were estimated via IPTW for the primary and secondary outcomes.

## Results

**Figure 1:**
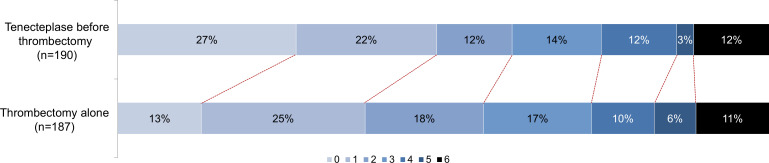
Study flow chart.

In accordance with the eligibility criteria of the target trial, 377 of 602 (62.6%) potentially eligible patients were included in the analysis. The main reasons for exclusion were an occlusion location other than the intracranial ICA or the M1 segment of the MCA, a primary presentation at a site without thrombectomy capability, no intention to perform thrombectomy, prestroke functional dependence (mRS>2), a low NIHSS on baseline (<5 points) and missing data for the primary outcome or variables used for weighting (figure 1). Additionally, two patients were excluded due to a non-random violation of the positivity assumption with a propensity score >0.73 (online supplemental appendix eFigure 2).

### Baseline characteristics

Of the 377 patients, 190 (50.4%) were treated with tenecteplase before thrombectomy and 187 (49.6%) patients received thrombectomy alone. Patients treated with thrombectomy alone had longer onset-to-randomisation times, lower baseline ASPECTS and less frequent atrial fibrillation at baseline. Other baseline characteristics were balanced between the two groups (table 2).

**Table 2:** Baseline characteristics

	Thrombectomy alone	Tenecteplase before thrombectomy
Patients, n	187	190
Demographics		
Age, years, median (IQR)	73 (64–81)	72 (63–80)
Male sex, n (%)	87 (46.5)	106 (55.8)
Stroke characteristics		
NIHSS, median (IQR)	17(13–20)	17(14–21)
Onset-to-randomisation time, min, median (IQR)	123 (99–166)	112 (87–145)
Onset-to-arterial puncture time, min, median (IQR)	152 (127–192)	152 (129–199)
IVT-to-arterial puncture time, min, median (IQR)	Not applicable	34 (20–50)
Occlusion location, n (%)		
ICA	56 (30.0)	50 (26.3)
Proximal M1	69 (36.9)	78 (41.1)
Distal M1	62 (33.1)	62 (32.6)
Tandem occlusion	28 (15.0)	30 (15.8)
ASPECTS*	8 (8 9)	9 (8–10)
Medical history		
Hypertension, n (%)	111 (61.0)	116 (61.1)
Diabetes mellitus, n (%)	22 (12.4)	34 (17.9)
Atrial fibrillation, n (%)	16 (9.1)	57 (30.0)
Prior ischaemic stroke, n (%)	21 (11.7)	26 (13.7)

* One point was added to ASPECTS scored using MRI if ASPECTS<10 to account for MRI-CT differences.

ASPECTS, Alberta Stroke Programme Early CT Score; ICA, internal carotid artery; IVT, intravenous thrombolysis; NIHSS, National Institutes of Health Stroke Scale.

### Tenecteplase before thrombectomy versus thrombectomy alone

**Figure 2:**
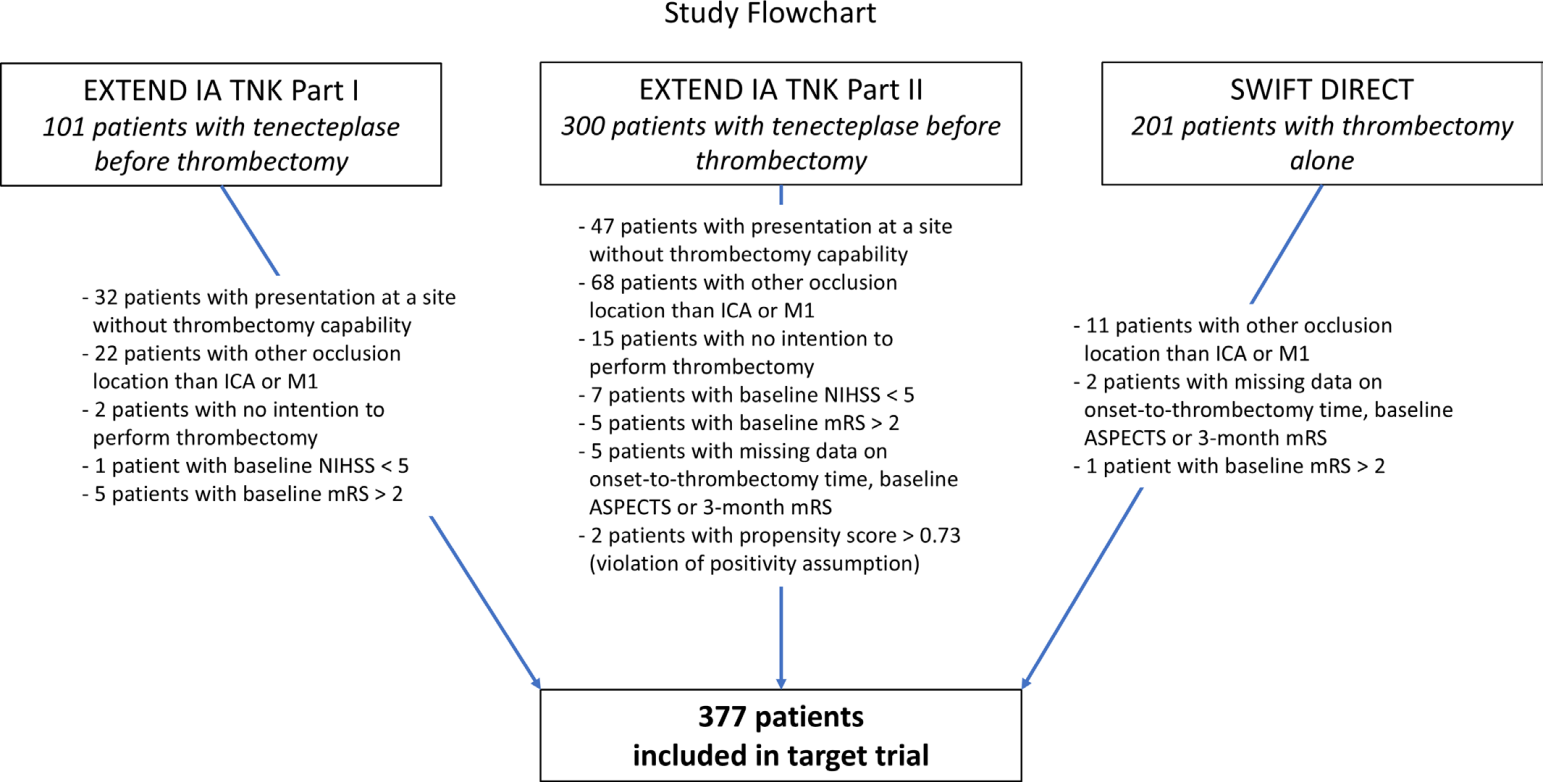
Distribution of the mRS at 90 days. ASPECTS, Alberta Stroke Programme Early CT Score; ICA, internal carotid artery; mRS, modified Rankin Scale; NIHSS, National Institutes of Health Stroke Scale; SWIFT DIRECT, Solitaire With the Intention for Thrombectomy Plus Intravenous t-PA vs DIRECT Solitaire StentRetriever Thrombectomy in Acute Anterior Circulation Stroke Trial.

At 90 days, 115 (60.5%) patients were functionally independent in the tenecteplase before thrombectomy group vs 105 (56.2%) in the thrombectomy alone group. This difference was not statistically significant after adjusting for potential confounders with identical results using both IPTW and G-Computation (SRD 0.04 (95% CI −0.06 to 0.13)). Among prespecified secondary outcomes, tenecteplase before thrombectomy resulted in significantly higher odds of functional improvement by at least one mRS category (IPTW acOR 1.56 (95% CI 1.07 to 2.23)) and increased the proportion with freedom from disability at 90 days (IPTW: SRD 0.10 (95% CI 0.01 to 0.20), G-Computation: SRD 0.10 (95% CI 0.007 to 0.20)). This corresponds to an estimated number needed to treat of approximately 10. Rates of reperfusion prior to thrombectomy were significantly higher in patients who received tenecteplase (12.1%) vs thrombectomy alone (0.5%). Tenecteplase before thrombectomy was not significantly associated with mortality or sICH in either the IPTW or G-Computation analyses (table 3, figure 2). In a sensitivity analysis restricted to patients who received 0.25 mg/kg tenecteplase before thrombectomy (119, 62.6%), effects on the primary and secondary outcomes were similar to the main analysis (table 4, online supplemental appendix eFigure 3). Within patients receiving tenecteplase, whether the time from tenecteplase to initiation of thrombectomy was less than or greater than 30 min made no observable difference to outcomes (online supplemental appendix eTable 2, eFigure 3).

**Table 3:** Clinical outcomes stratified by treatment

	Tenecteplase before thrombectomy	Thrombectomy alone	IPTW Tenecteplase before Thrombectomy vs thrombectomy alone*	G computation tenecteplase before thrombectomy vs thrombectomy alone*
Functional independence†	115 (60.05)	105 (56.2)	SRD 0.04 (–0.06, 0.13) aOR 1.21 (0.77, 1.91)	SRD 0.04 (−0.06, 0.13)
Functional improvement (ordinal mRS)‡	2 (0–4)	2(1–4)	**acOR 1.56 (1.07, 2.23**)	Not applicable
Freedom from disability§	93 (48.9)	71 (38.0)	**SRD 0.10 (0.01, 0.20) aOR 1.61 (1.03, 2.50**)	**SRD 0.10 (0.007, 0.20**)
Mortality	22 (11.6)	21 (11.2)	SRD −0.003 (−0.06, 0.06) aOR 0.92 (0.46, 1.87)	SRD −0.003 (−0.06, 0.06)
sICH	7 (3.7)	3 (1.6)	SRD 0.02 (-0.01–0.05) aOR 2.50 (0.60–10.4)	SRD 0.02 (−0.01, 0.06)
Reperfusion prior to thrombectomy¶	23 (12.1)	1 (0.5)	**SRD 0.11 (0.06–0.15**)	**SRD 0.17 (0.05–0.28**)

Bold values: p < 0.05

* Adjusted for age, pretreatment NIHSS, clot location, baseline ASPECTS, onset-to-arterial-puncture time.

† Defined as mRS 0–2.

‡ Data are represented as median (IQR).

§ Defined as mRS 0–1.

¶ Defined as cross-sectional eTICI score of 2b–3 (>50% reperfusion of the involved vascular territory) on the first run of digital subtraction angiography.

a(c)OR, adjusted (common) OR; ASPECTS, Alberta Stroke Programme Early CT Score; eTICI, expanded Thrombolysis in Cerebral Infarction; IPTW, inverse probability treatment weighting; mRS, modified Rankin Scale; NIHSS, National Institutes of Health Stroke Scale; sICH, symptomatic intracranial haemorrhage; SRD, standardised risk difference.

**Table 4:** Clinical outcomes stratified by treatment and dose of tenecteplase

	Tenecteplase 0.25 mg/kg			Tenecteplase 0.40 mg/kg		
	Tenecteplase before thrombectomy n=119	Thrombectomy alone n=187	IPTW tenecteplase before thrombectomy vs thrombectomy alone*	Tenecteplase before thrombectomy n=71	Thrombectomy alone n=187	IPTW tenecteplase before thrombectomy vs thrombectomy alone*
Functional independence†	74 (62.2)	105 (56.2)	SRD 0.04 (–0.06, 0.15) aOR 1.29 (0.76, 2.19)	41 (57.8)	105 (56.2)	SRD 0.01 (–0.12, 0.14) aOR 1.08 (0.59, 1.96)
mRS score reduction (shift analysis)‡	1 (0–4)	2(1–4)	**acOR 1.62 (1.06, 2.47**)	2(1–3)	2(1–4)	acOR 1.45 (0.89, 2.38)
Freedom of disability§	60 (50.4)	71 (38.0)	**SRD 0.11 (0.0004, 0.22) aOR 1.72 (1.04, 2.85**)	33 (46.5)	71 (38.0)	SRD 0.06 (–0.07, 0.19) aOR 1.41 (0.78, 2.54)
Mortality	13 (10.9)	21 (11.2)	SRD −0.007 (–0.08, 0.06) aOR 0.89 (0.40, 2.00)	9 (12.7)	21 (11.2)	SRD 0.003 (–0.08, 0.09) aOR 0.90 (0.37, 2.21)
sICH	3 (2.5)	3 (1.6)	SRD 0.02 (–0.02, 0.05) aOR 1.93 (0.42, 8.94)	4 (5.6)	3 (1.6)	SRD 0.04 (–0.02, 0.10) aOR 3.57 (0.64, 20.09)

Bold values: p < 0.05

* Adjusted for age, baseline NIHSS, clot location, adjusted baseline ASPECTS, onset-to-arterial-puncture time.

† Defined as mRS 0–2.

‡ Data represented as median (IQR).

§ Defined as mRS 0–1.

a(c)OR, adjusted (common) odds; ASPECTS, Alberta Stroke Programme Early CT Score; IPTW, inverse probability treatment weighting; mRS, modified Rankin Scale; NIHSS, National Institutes of Health Stroke Scale; sICH, symptomatic intracranial haemorrhage; SRD, standardixed risk difference.

### Association of (expected) onset-to-thrombolytic time with outcomes

In patients receiving tenecteplase before thrombectomy, 130 (68.4%) had an actual onset-to-intravenous thrombolysis (IVT) time ≤140 min and in the thrombectomy-alone group 106 (56.7%) patients had an expected onset-to-IVT time ≤140 min. In the subgroup with (expected) onset-to-thrombolytic time ≤140 min, tenecteplase before thrombectomy did not increase the rate of functional independence at 90 days (SRD 0.08 (95% CI −0.04 to 0.19)) but resulted in improved outcomes in the ordinal analysis of the mRS (acOR 1.63 (95% CI 1.04 to 2.56)) and an increased probability for freedom of disability (SRD 0.15 (95% CI 0.02 to 0.27)) at 90 days. In patients with an (expected) onset-to-thrombolytic time >140 min, tenecteplase before thrombectomy was not associated with any outcome (table 5, online supplemental appendix eFigures 3,4). No significant treatment-by-(expected) onset-to-thrombolytic time interaction was observed in all outcomes (table 5). Investigating other (expected) onset-to-thrombolytic time thresholds, the probability for improvement of outcomes over the full mRS scale increased at 120 min (acOR 1.76 (95% CI 1.03 to 3.01)) and decreased at 180 min (acOR 1.49 (95% CI 0.99 to 2.23)) compared with the prespecified threshold of 140 min (online supplemental appendix eTable 3).

**Table 5:** Clinical outcomes stratified by treatment and expected onset-to-thrombolytic time

	Expected onset-to-thrombolytic time ≤140 min			Expected onset-to-thrombolytic time >140 min			
	Tenecteplase before thrombectomy n=130	Thrombectomy alone n=106	IPTW Tenecteplase before thrombectomy vs thrombectomy alone*	Tenecteplase before thrombectomy n=60	Thrombectomy alone n=81	IPTW Tenecteplase before thrombectomy vs thrombectomy alone	Interaction p value
Functional independence†	84 (64.6)	62 (58.5)	SRD 0.08 (–0.04, 0.19) aOR 1.41 (0.78, 2.54)	31 (51.7)	43 (53.1)	SRD 0.0006 (–0.17, 0.17) aOR 0.89 (0.43, 1.82)	0.30
Functional improvement (ordinal mRS)‡	1 (0–3)	2(1–3)	**acOR 1.63 (1.04, 2.56**)	2(1–4)	2(1–4)	acOR 1.23 (0.67, 2.28)	0.46
Freedom from disability§	70 (53.9)	43 (40.6)	**SRD 0.15 (0.02, 0.27) aOR 1.80 (1.03, 3.16**)	23 (38.3)	28 (34.6)	SRD 0.03 (–0.14, 0.20) aOR 1.18 (0.56, 2.46)	0.34
Mortality	15 (11.5)	8 (7.6)	SRD 0.02 (–0.04, 0.09) aOR 1.38 (0.44, 4.30)	7 (11.7)	13 (16.1)	SRD −0.06 (–0.18, 0.05) aOR 0.61 (0.20, 1.85)	0.24
Reperfusion prior to thrombectomy	13 (10.0)	1 (0.9)	**SRD 0.08 (0.03, 0.13**)	10 (16.7)	0 (0)	Not estimable¶	Not applicable

Expected thrombolytic time for thrombectomy-along group calculated by adding the mean time from randomisation to IVT (derived from patients receiving bridging thrombolysis with alteplase in the SWIFT DIRECT trial) to the time from onset to randomisation of each patient.

Bold values: p < 0.05

* Adjusted for age, baseline NIHSS, clot location, adjusted baseline ASPECTS.

† Defined as mRS 0–2.

‡ Data represented as median (IQR).

§ Defined as mRS 0–1.

¶ Due to low event rates.

a(c)OR, adjusted (common) OR; ASPECTS, Alberta Stroke Programme Early CT Score; IPTW, inverse probability treatment weighting; IVT, intravenous thrombolysis; mRS, modified Rankin Scale; NIHSS, National Institutes of Health Stroke Scale; SRD, standardised risk difference; SWIFT DIRECT, Solitaire With the Intention for Thrombectomy Plus Intravenous t-PA vs DIRECT Solitaire StentRetriever Thrombectomy in Acute Anterior Circulation Stroke Trial.

## Discussion

In this target trial emulation of individual patient data from three randomised controlled trials comparing tenecteplase before thrombectomy versus thrombectomy alone, we found that tenecteplase before thrombectomy did not impact functional independence at 90 days when compared with thrombectomy alone but resulted in increased rates of freedom from disability and functional improvement by at least one category in ordinal analysis of the full mRS scale at 90 days. The improvement in outcomes was most evident in patients treated early with tenecteplase (time-to-IVT≤140 min). Finally, rates of reperfusion prior to thrombectomy were higher in patients receiving tenecteplase before thrombectomy, while safety outcomes did not significantly differ compared with patients treated with thrombectomy alone.

In the Improving Reperfusion Strategies in Acute Ischaemic Stroke (IRIS) collaboration meta-analysis of six randomised controlled trials, thrombectomy alone was neither inferior nor non-inferior compared with bridging thrombolysis before thrombectomy in patients with stroke due to anterior circulation large-vessel occlusion.^5^ However, these findings primarily apply to bridging thrombolytic with alteplase. Randomised controlled trials investigating bridging with tenecteplase versus thrombectomy alone are underway (BRIDGE-TNK, NCT04733742; DIRECT-TNK, NCT05199194) but the estimated completion date for these trials is March 2026 and July 2027, respectively.

Currently, there is a widespread transition to the use of tenecteplase in clinical practice, based on the demonstration of superiority over alteplase in a meta-analysis,^27^ guideline changes,^28^,^30^ regulatory approval in some jurisdictions^31^ and the practical advantages of bolus administration. Our study, therefore, provides a timely insight into the potential clinical impact of tenecteplase as a bridging thrombolytic. In our analysis, the incidence of the primary outcome was similar in both treatment groups, but bridging with tenecteplase resulted in statistically significant and clinically meaningful improvements in the secondary functional outcomes. The number needed to treat to achieve disability-free recovery was approximately 10 for patients treated with the standard dose of tenecteplase (0.25 mg/kg). In addition, rates of reperfusion prior to thrombectomy were substantially higher in patients receiving tenecteplase before thrombectomy compared with thrombectomy alone. Reperfusion prior to thrombectomy has been associated with reduced disability, consistent with the strong links between time to reperfusion and outcome.^32^ The rate of reperfusion prior to thrombectomy in patients receiving alteplase in the IRIS metanalysis was lower compared with patients treated with tenecteplase in our target trial (4.0% vs 12.1%). This is in line with a recent meta-analysis investigating outcomes in patients receiving alteplase versus tenecteplase before thrombectomy that found significantly higher rates of reperfusion prior to thrombectomy in patients treated with tenecteplase.^33^ It is important to note that the median thrombolytic-to-thrombectomy time in our target trial was longer compared with the IRIS meta-analysis (25 min vs 34 min), increasing the time with active thrombolysis before thrombectomy. This difference is also indirectly reflected in the baseline characteristics of our target trial: the time from onset to randomisation was shorter in patients who received tenecteplase before thrombectomy, whereas the onset-to-thrombectomy time was similar in both groups. Other imbalances in baseline characteristics were that patients treated with thrombectomy only had a lower baseline ASPECTS and were less likely to have a history of atrial fibrillation. While the possible causes of these differences remain speculative, it is noteworthy that ASPECTS was included for weighting and adjusting in the statistical analyses and that a recent meta-analysis showed no association between atrial fibrillation and outcomes in patients treated with thrombectomy.^34^

The previously performed TIMELESS trial^24^ partially resembled our target trial given the high proportion (397/458, 87%) of patients with a large-vessel occlusion anterior circulation stroke who were planned to receive thrombectomy after treatment with tenecteplase or placebo. In this subgroup of TIMELESS, the point estimate for functional improvement (acOR 1.20 (95% CI 0.85 to 1.70)) favoured bridging thrombolytic with tenecteplase without reaching statistical significance. However, this trial differed from our target trial as it enrolled patients in the extended time window (4.5–24 hours after last known well), included patients with occlusion of the M2 segment of the MCA and had very short (median 15 min) thrombolytic to arterial puncture times, which might explain the different results.

When only analysing patients receiving bridging with tenecteplase with an onset-to-tenecteplase time within 140 min (vs patients with an expected onset-to-tenecteplase time within 140 min in the thrombectomy alone group) the effect size estimate for freedom from disability and improvement of the mRS over the full ordinal scale at 90 days increased compared with our main analysis. Conversely, no trend towards improved functional outcomes was observed in patients receiving bridging with tenecteplase with an onset-to-tenecteplase time>140 min (vs patients with an expected onset-to-tenecteplase time >140 min in the thrombectomy alone group), although tests of interaction between treatment and onset-to-tenecteplase time were not significant, possibly due to the limited sample size. Also taking into account the gradually increasing probability for improvement of outcomes with decreasing time from onset to (expected) administration of tenecteplase (from 120 to 180 min), our findings corroborate data derived from patients treated with alteplase^22^ and suggest that administering tenecteplase as early as possible after stroke onset (perhaps ideally prehospital in a mobile stroke unit)^35 36^ is likely to maximise treatment benefit. Interestingly, the increase in early reperfusion when intravenous thrombolytic was commenced earlier observed in the EXTEND-IA TNK trials,^37^ and associated with improved functional outcome, was not evident when dichotomised at 140 min.

When stratifying the tenecteplase before thrombectomy group by the time between thrombolytic and initiation of thrombectomy (within or after 30 min), no clear differences in outcomes were observed. This contrasted with an observational study that suggested worse outcomes in patients with increased time from thrombolytic to thrombectomy^23^ but may relate to our patients all presenting directly to thrombectomy-capable centres, with few patients having a long delay from arrival to thrombectomy, and the longer half-life of tenecteplase. On the other side, the median dwell time of 19 min in the subgroup of patients with a thrombolytic-to-arterial-puncture time within 30 min seems to have been sufficient to still enable high rates of early reperfusion (9.2%).

There are several limitations to this study. Despite emulating a target trial and adjusting for potential confounders, a risk of ascertainment bias remains as the two treatment arms of our target trial originate from different randomised controlled trials, each conducted at a specific time point and within a specific setting. Thus, some treatment group differences may remain unbalanced as adjustment was not feasible. Additionally, although applying the target trial eligibility criteria some baseline imbalances remained in our analysed data which might have confounded our results. Due to the sample size, our subgroup analyses are likely underpowered. Hence, the subanalysis investigating expected onset-to-thrombolytic time was dichotomised at 140 min, based on prior studies, and we were not able to evaluate outcomes over the full range of the onset-to-thrombolytic time. Compared with the ongoing randomised controlled trials BRIDGE-TNK and DIRECT-TNK (with estimated sample sizes of 544 and 390 patients, respectively), the sample size of our target study (377 patients) was rather small, possibly leading to a neutral primary outcome and limiting overall generalisability. Our data included only patients presenting directly to thrombectomy-capable centres. Other trials support the benefits of tenecteplase in patients with large-vessel occlusion who present to centres without on-site thrombectomy, including in an extended time window.^13 38^

## Conclusion

This causal inference study using target trial emulation of individual patient data from three randomised controlled trials showed that tenecteplase as bridging thrombolytic before thrombectomy did not result in increased odds of functional independence when compared with thrombectomy alone. However, tenecteplase before thrombectomy improved functional outcomes over the full mRS scale and increased freedom from disability at 90 days. These findings remained unchanged in a sensitivity analysis including only patients treated with 0.25 mg/kg tenecteplase. The benefit of tenecteplase appeared most evident in patients treated early after stroke onset. Tenecteplase is now guideline-recommended and, in some cases, labelled for stroke in multiple jurisdictions. A recent meta-analysis has demonstrated superiority over alteplase for a broad range of thrombolytic-eligible patients with ischaemic stroke.^27^ Pending dedicated randomised trials, our target trial emulation provides support for the use of tenecteplase in thrombolytic-eligible patients 0–4.5 hours after onset of stroke due to large-vessel occlusion who are planned to receive endovascular thrombectomy.

## Supplementary material

10.1136/jnnp-2024-335325 online supplemental file 1

## Data Availability

Data may be obtained from a third party and are not publicly available.
