# Supplementary material for: Bridging thrombolysis with tenecteplase versus endovascular thrombectomy alone for large-vessel anterior circulation stroke: a target trial emulation analysis
Source: J Neurol Neurosurg Psychiatry. 2025 Jan 22;96(8):e335325. doi: 10.1136/jnnp-2024-335325 (PMC12322409; doi:10.1136/jnnp-2024-335325)
Supplement: online supplemental file 1 [file jnnp-96-8-s001.pdf]

Supplemental Material

Table of Content

**Supplementary Tables**	**p 2-4**
eTable 1. Characteristics of the included trials.	**p 2**
eTable 2. Clinical Outcomes stratified by Treatment and Bridging-to-thrombectomy time.	**p 3**
eTable 3. Clinical Outcomes stratified by Treatment and expected onset-to-thrombolytic time.	**p 4**
**Supplementary Figures**	**p 5-8**
eFigure 1. Directed acyclic graphs.	**p 5**
eFigure 2. Boxplot of the propensity scores stratified by treatment.	**p 6**
eFigure 3. Forest plots for odds ratios of clinical outcomes in subgroups.	**p 7**
eFigure 4. mRS Scores at 90 Days Stratified by Treatment and onset-to-tenecteplase time.	**p 8**
**Supplemental Methods**	**p 9-20**
eMethods 1. Strengthening the Reporting of Observational Studies in Epidemiology (STROBE) reporting guideline	**p 10-13**
eMethods 2. Pre-specified statistical Analysis Plan.	**p 14-18**
eMethods 3. Rationale and methods for subgroup "Time from tenecteplase to thrombectomy"	**p 19**
**List of investigators**	**p 20-22**

Supplementary Tables

eTable 1. Characteristics of the included trials.

Characteristics	SWIFT DIRECT	EXTEND-IA TNK	EXTEND-IA TNK Part 2
Main inclusion criteria	Age ≥18 years,<br>Baseline mRS 0-1,<br>ICA/M1: CTA/MRA,<br>NIHSS ≥5/<30,<br>Imaging: ASPECTS: DWI/CT ≥4,<br>Onset-randomisation ≤ 4 hours 15 min	Age ≥18 years,<br>Baseline mRS 0-3,<br>ICA/M1/M2/BA: CTA/MRA,<br>NIHSS: not required,<br>Imaging: Hypodensity < 1/3 MCA/ BA territory,<br>Onset-to-IVT: ≤ 4 hours 30 min	Age ≥18 years,<br>Baseline mRS 0-3,<br>ICA/M1/M2/BA: CTA/MRA,<br>NIHSS: not required,<br>Imaging: Hypodensity < 1/3 MCA/ BA territory,<br>Onset-to-IVT: ≤ 4 hours 30 min,
Primary outcome	mRS 0-2 at 90 days	Reperfusion of greater than 50% of the involved ischaemic territory or an absence of retrievable thrombus	Reperfusion of greater than 50% of the involved ischaemic territory
Treatment	EVT (n=201)<br>IVT (alteplase) + EVT (n=207)	IVT (alteplase) + EVT (n=101)<br>IVT (tenecteplase) + EVT (n=101)	IVT (tenecteplase) + EVT (n=150)<br>IVT (tenecteplase) + EVT (n=150)

Abbreviations: mRS = modified Rankin Scale, ICA = internal carotid artery, M1 = M1 segment of the middle cerebral artery, M2 = M2 segment of the middle cerebral artery, BA = basilar artery, NIHSS = National Institutes of Health Stroke Scale, MCA = middle cerebral artery, CTA = Computed Tomography Angiography, Magnetic Resonance Angiography, ASPECTS = Alberta Stroke Program Early CT Score, DWI = Diffusion-weighted imaging, EVT = endovascular thrombectomy, intravenous thrombolysis

eTable 2. Clinical Outcomes stratified by Treatment and Time from Tenecteplase to Thrombectomy.

	**Time from Tenecteplase to Thrombectomy ≤ 30min** (median [IQR] 19[12-23] min)			**Time from Tenecteplase to Thrombectomy > 30min** (median [IQR] 48[39-60] min)		
	**Tenecteplase before Thrombectomy** n = 87	**Thrombectomy alone** n = 187	**IPTW Tenecteplase before Thrombectomy vs Thrombectomy alone[1]**	**Tenecteplase before Thrombectomy** n = 103	**Thrombectomy alone** n = 187	**IPTW Tenecteplase before Thrombectomy vs Thrombectomy alone[1]**
Functional independence[2]	55 (63.2)	105 (56.2)	SRD 0.03 (-0.09 – 0.15) aOR 1.13 (0.64 – 2.02)	60 (58.3)	105 (56.2)	SRD 0.02 (-0.10 – 0.13) aOR 1.07 (0.61 – 1.86)
Functional Improvement (ordinal mRS analysis)[3]	1 [0-4]	2 [1-4]	acOR 1.52 (0.86 – 2.17)	2 [1-3]	2 [1-4]	acOR 1.54 (0.98 – 2.41)
Freedom from disability[4]	47 (54.0)	71 (38.0)	**SRD 0.13 (0.001 – 0.25)** aOR 1.71 (0.99 – 2.96)	46 (44.7)	71 (38.0)	SRD 0.05 (-0.07 – 0.17) aOR 1.30 (0.76 – 2.24)
Mortality	12 (13.8)	21 (11.2)	SRD 0.04 (-0.04 – 0.12) aOR 1.69 (0.63 – 4.50)	10 (9.7)	21 (11.2)	SRD -0.02 (-0.09 – 0.05) aOR 0.86 (0.36 – 2.05)
Reperfusion prior to thrombectomy	8 (9.2)	1 (0.5)	**0.10 (0.03-0.16)**	15 (14.6)	1 (0.5)	**0.13 (0.06-0.19)**

Abbreviations: IPTW = Inverse probability treatment weighting, SRD = standardized risk difference, a(c)OR = adjusted (common) odds ratio with 95% confidence intervals, mRS = modified Rankin Scale
1 adjusted for Age, baseline NIHSS, clot location, adjusted baseline ASPECTS, onset-to-arterial-puncture time
2 Defined as mRS 0-2
3 Data represented as median [interquartile range]
4 Defined as mRS 0-1

eTable 3. Clinical Outcomes stratified by Treatment and expected onset-to-thrombolytic time.

	**Expected onset-to-thrombolytic time ≤ 120min**			**Expected onset-to-thrombolytic time ≤ 140min**			**Expected onset-to-thrombolytic time ≤ 180min**		
	**Tenecteplase before Thrombectomy** n = 101	**Thrombectomy alone** n = 69	**IPTW Tenecteplase before Thrombectomy vs Thrombectomy alone**[1]	**Tenecteplase before Thrombectomy** n = 130	**Thrombectomy alone** n = 106	**IPTW Tenecteplase before Thrombectomy vs Thrombectomy alone**[1]	**Tenecteplase before Thrombectomy** n = 160	**Thrombectomy alone** n = 143	**IPTW Tenecteplase before Thrombectomy vs Thrombectomy alone**
Functional independence[2]	68 (68.0)	44 (63.8)	aOR 1.25 (0.61 – 2.56)	84 (64.6)	62 (58.5)	aOR 1.41 (0.78 – 2.54)	99 (61.9)	83 (58.0)	aOR 1.25 (0.74 – 2.10)
Functional improvement (ordinal mRS)[3]	1 [0-3]	2 [1-3]	**acOR 1.76 (1.03 – 3.01)**	1 [0-3]	2 [1-3]	**acOR 1.63 (1.04 – 2.56)**	1 [0-4]	2 [1-4]	acOR 1.49 (0.99 – 2.23)
Freedom from disability[4]	56 (55.5)	27 (39.1)	**aOR 2.00 (1.01 – 3.96)**	70 (53.9)	43 (40.6)	**aOR 1.80 (1.03 – 3.16)**	81 (50.6)	55 (38.5)	**aOR 1.66 (1.01 – 2.73)**
Mortality	11 (10.9)	5 (7.3)	aOR 0.88 (0.21 – 3.63)	15 (11.5)	8 (7.6)	aOR 1.38 (0.44 – 4.30)	18 (11.3)	12 (8.4)	aOR 1.10 (0.44 – 2.76)

Expected thrombolytic time for thrombectomy-along group calculated by adding the mean time from randomisation to IVT (derived from patients receiving bridging thrombolysis with alteplase in the SWIFT DIRECT trial) to the time from onset to randomisation of each patient.

Abbreviations: IPTW = Inverse probability treatment weighting, a(c)OR = adjusted (common) odds ratio, mRS = modified Rankin Scale

[1] adjusted for Age, baseline NIHSS, clot location, adjusted baseline ASPECTS

[2] Defined as mRS 0-2

[3] Data represented as median [interquartile range]

[4] Defined as mRS 0-1

Supplementary Figures

eFigure 1. Directed acyclic graphs.

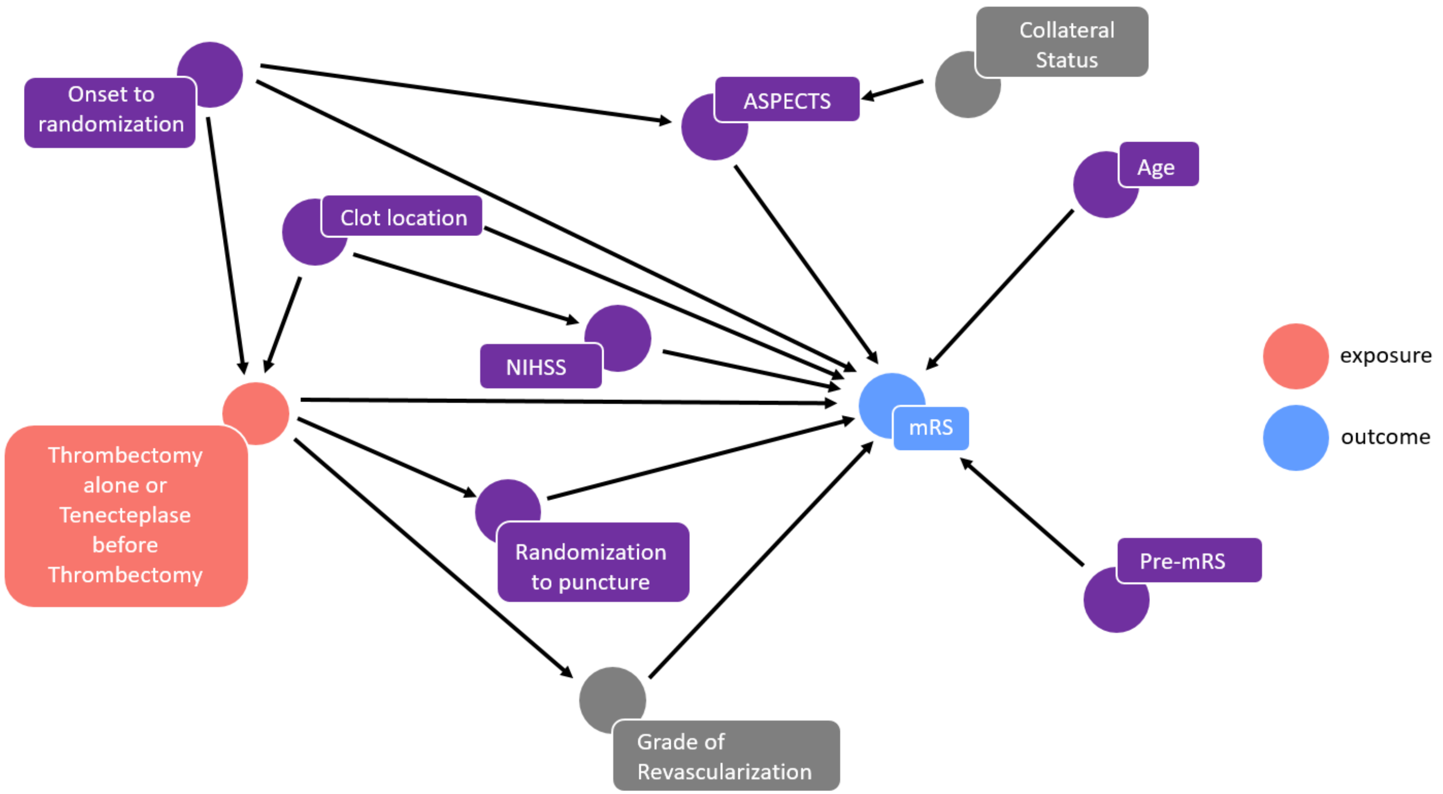

Variables highlighted in purple were accounted for by inclusion in models and/ or by inclusion in eligibility criteria for the target trial.

eFigure 2. Boxplot of the propensity scores stratified by treatment.

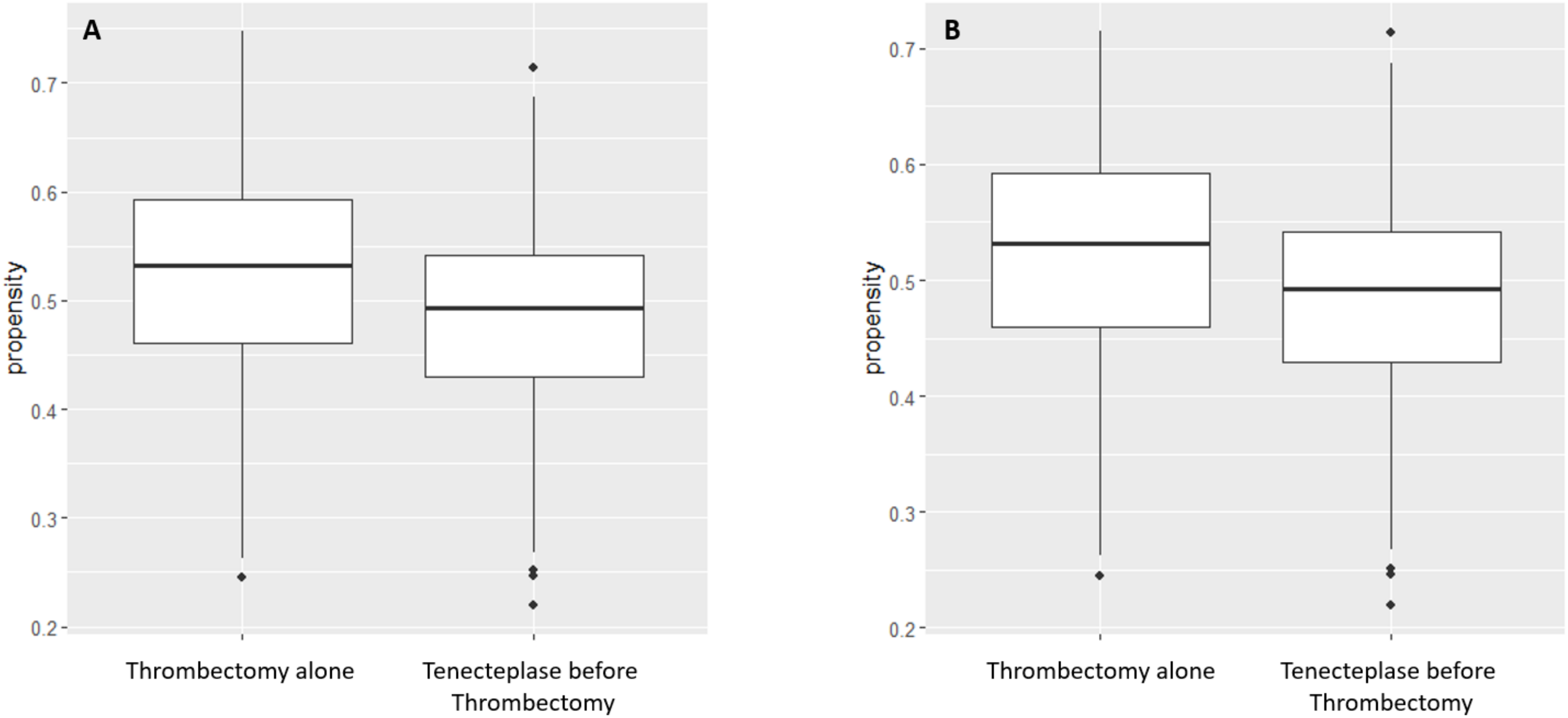

A Before exclusion of patients with propensity > 0.73
B After exclusion of patients with propensity > 0.73

eFigure 3. Forest plots for odds ratios of clinical outcomes in subgroups.

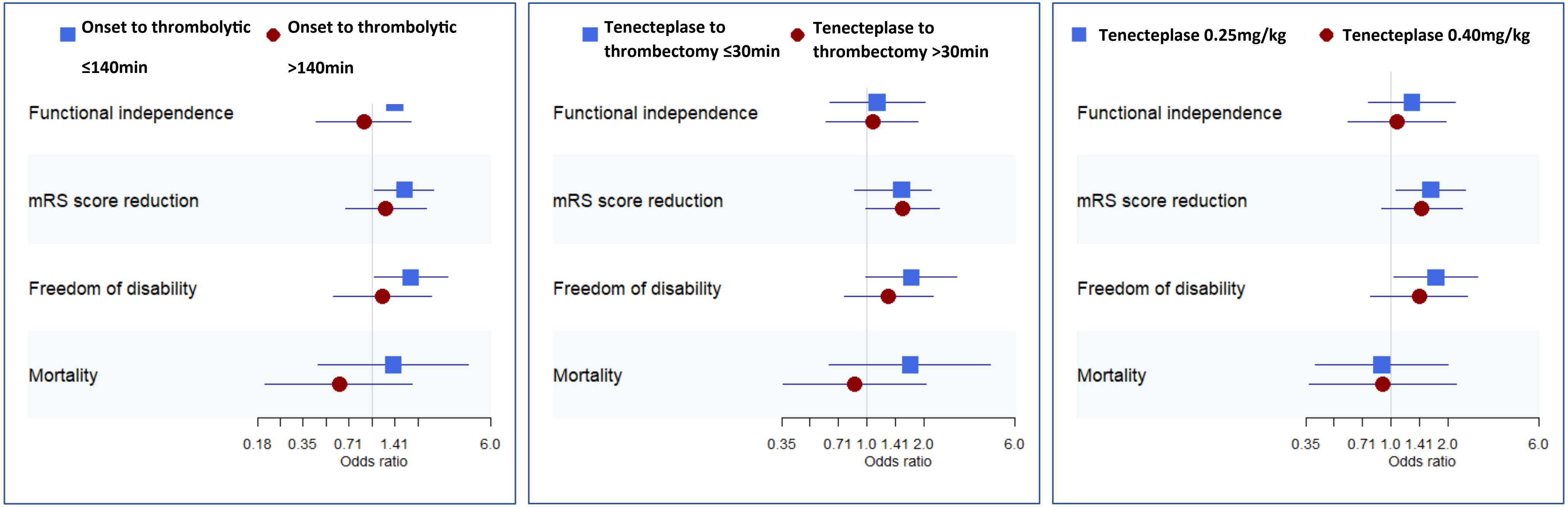

eFigure 4. mRS Scores at 90 Days Stratified by Treatment and onset-to-thrombolytic time.

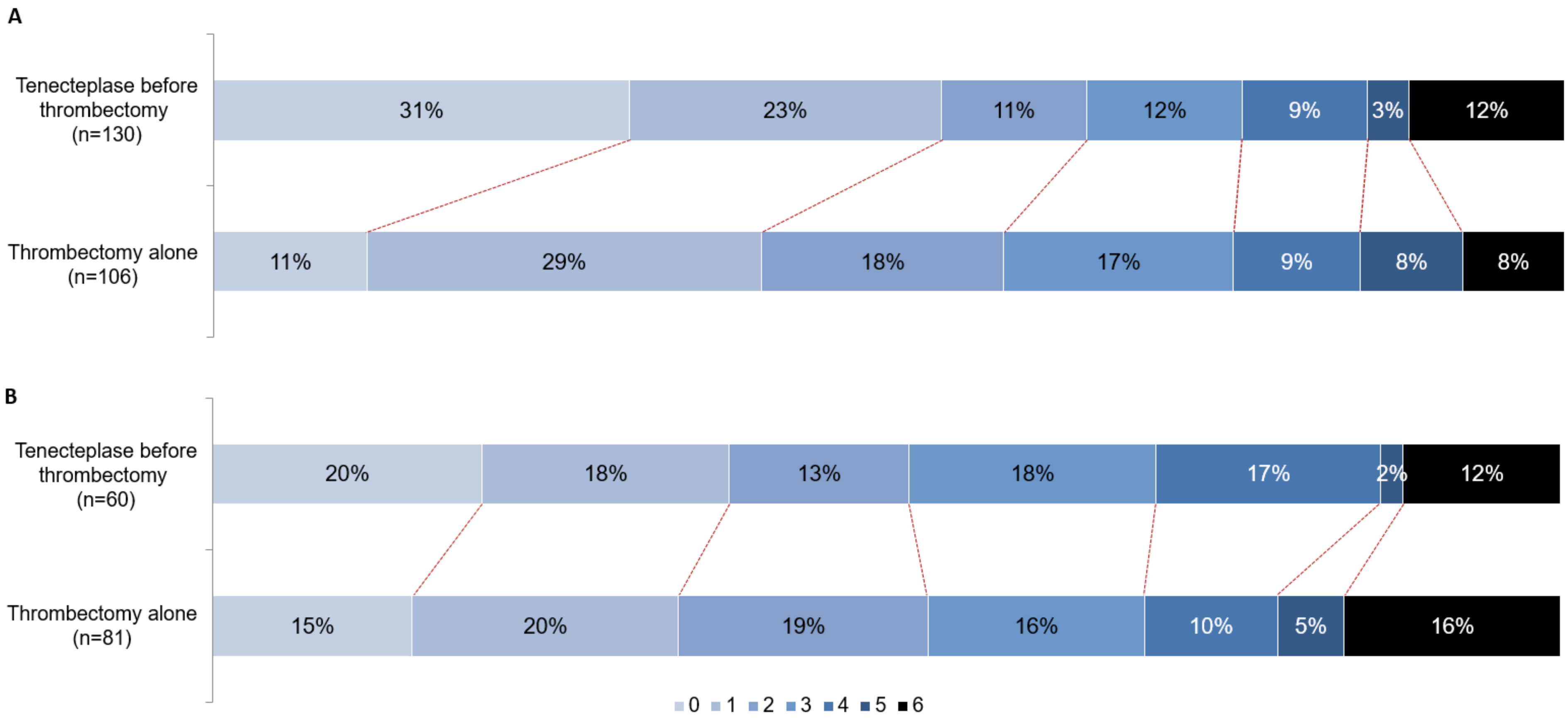

A Patients with (expected) onset-to-thrombolytic time ≤ 140 minutes
B Patients with (expected) onset-to-thrombolytic time > 140 minutes

Supplemental Methods

eMethods 1. STROBE Statement—checklist of items that should be included in reports of observational studies

	Item No.	Recommendation	Page No.	Relevant text from manuscript
**Title and abstract**	1	(*a*) Indicate the study's design with a commonly used term in the title or the abstract	3	This is a causal inference study of observational data
		(*b*) Provide in the abstract an informative and balanced summary of what was done and what was found	3	Tenecteplase before thrombectomy compared to thrombectomy alone did not increase the probability of functional independence
**Introduction**				
Background/rationale	2	Explain the scientific background and rationale for the investigation being reported	5	Randomised controlled data in patients treated with tenecteplase prior to thrombectomy versus thrombectomy alone are currently lacking
Objectives	3	State specific objectives, including any prespecified hypotheses	5	Therefore, we compared bridging thrombolytic with tenecteplase before thrombectomy
**Methods**				
Study design	4	Present key elements of study design early in the paper	6	For this analysis, we emulated a hypothetical target trial
Setting	5	Describe the setting, locations, and relevant dates, including periods of recruitment, exposure, follow-up, and data collection	6	We used data from three randomised controlled trials

Participants	6	(*a*) *Cohort study*—Give the eligibility criteria, and the sources and methods of selection of participants. Describe methods of follow-up<br><br>*Case-control study*—Give the eligibility criteria, and the sources and methods of case ascertainment and control selection. Give the rationale for the choice of cases and controls<br><br>*Cross-sectional study*—Give the eligibility criteria, and the sources and methods of selection of participants	6	Target trial inclusion criteria were
		(*b*) *Cohort study*—For matched studies, give matching criteria and number of exposed and unexposed<br><br>*Case-control study*—For matched studies, give matching criteria and the number of controls per case	N/A	
Variables	7	Clearly define all outcomes, exposures, predictors, potential confounders, and effect modifiers. Give diagnostic criteria, if applicable	7-8	The primary outcome was functional independence
Data sources/ measurement	8*	For each variable of interest, give sources of data and details of methods of assessment (measurement). Describe comparability of assessment methods if there is more than one group	8	Images were graded by the respective trial core lab
Bias	9	Describe any efforts to address potential sources of bias	8-9	Subsequently, we estimated the average causal treatment effect
Study size	10	Explain how the study size was arrived at	6<br><br>12	We used data from three randomised controlled trials …<br><br>In accordance with the eligibility criteria of the target trial, 377 of 602

Continued on next page

Quantitative variables	11	Explain how quantitative variables were handled in the analyses. If applicable, describe which groupings were chosen and why	9-10	For IPTW, we fitted a logistic regression model of the exposure tenecteplase before thrombectomy as a function of the following covariables (according to the DAGs): age,
Statistical methods	12	(*a*) Describe all statistical methods, including those used to control for confounding	9-11	primary and secondary outcomes by using standardisation to population via:<br><br>a) Inverse probability of treatment weighting (IPTW) including previously identified covariates (i.e. modelling the treatment) and<br><br>b) G-Computation approach (i.e. modelling the outcome)
		(*b*) Describe any methods used to examine subgroups and interactions	11	To investigate a previously described time dependent effect
		(*c*) Explain how missing data were addressed	9	Cases with missing data for the primary outcome or confounding variables according to the DAGs were excluded
		(*d*) *Cohort study*—If applicable, explain how loss to follow-up was addressed<br><br>*Case-control study*—If applicable, explain how matching of cases and controls was addressed<br><br>*Cross-sectional study*—If applicable, describe analytical methods taking account of sampling strategy	9	Cases with missing data for the primary outcome or confounding variables according to the DAGs were excluded
		(*e*) Describe any sensitivity analyses	10	we performed a pre-specified subgroup sensitivity analysis

**Results**				
Participants	13*	(a) Report numbers of individuals at each stage of study—eg numbers potentially eligible, examined for eligibility, confirmed eligible, included in the study, completing follow-up, and analysed	12	In accordance with the eligibility criteria of the target trial, 377 of 602
		(b) Give reasons for non-participation at each stage	12	The main reasons for exclusion were
		(c) Consider use of a flow diagram		Figure 1
Descriptive data	14*	(a) Give characteristics of study participants (eg demographic, clinical, social) and information on exposures and potential confounders	12	Patients treated with thrombectomy alone had longer onset-to-randomisation times
		(b) Indicate number of participants with missing data for each variable of interest		Figure 1
		(c) *Cohort study*—Summarise follow-up time (eg, average and total amount)	N/A	
Outcome data	15*	*Cohort study*—Report numbers of outcome events or summary measures over time	12-13	At 90 days, 115 (60.5%) patients were functionally independent
		Case-control study—Report numbers in each exposure category, or summary measures of exposure	N/A	
		Cross-sectional study—Report numbers of outcome events or summary measures	N/A	
Main results	16	(*a*) Give unadjusted estimates and, if applicable, confounder-adjusted estimates and their precision (eg, 95% confidence interval). Make clear which confounders were adjusted for and why they were included	12-13	This difference was not statistically significant after adjusting<br><br>Table 3 and Table 4
		(*b*) Report category boundaries when continuous variables were categorized	13	130 (68.4%) had an actual onset-to-IVT time ≤140 minutes
		(*c*) If relevant, consider translating estimates of relative risk into absolute risk for a meaningful time period	15	to treat to achieve disability-free recovery was approximately 10

Continued on next page

Other analyses	17	Report other analyses done—eg analyses of subgroups and interactions, and sensitivity analyses	13	In sensitivity analysis restricted to patients who received 0.25mg/kg
**Discussion**				
Key results	18	Summarise key results with reference to study objectives	14	we found that tenecteplase before thrombectomy was not associated with functional independence
Limitations	19	Discuss limitations of the study, taking into account sources of potential bias or imprecision. Discuss both direction and magnitude of any potential bias	17	There are several limitations to this study. Despite emulating a target trial
Interpretation	20	Give a cautious overall interpretation of results considering objectives, limitations, multiplicity of analyses, results from similar studies, and other relevant evidence	17-18	This target trial emulation of individual patient data from three randomised controlled trials showed
Generalisability	21	Discuss the generalisability (external validity) of the study results	18	Pending dedicated randomised trials
**Other information**				
Funding	22	Give the source of funding and the role of the funders for the present study and, if applicable, for the original study on which the present article is based	12	The funders had no role in the design

*Give information separately for cases and controls in case-control studies and, if applicable, for exposed and unexposed groups in cohort and cross-sectional studies.

Note: An Explanation and Elaboration article discusses each checklist item and gives methodological background and published examples of transparent reporting. The STROBE checklist is best used in conjunction with this article (freely available on the Web sites of PLoS Medicine at http://www.plosmedicine.org/, Annals of Internal Medicine at http://www.annals.org/, and Epidemiology at http://www.epidem.com/). Information on the STROBE Initiative is available at www.strobe-statement.org.

eMethods 2. Pre-specified statistical Analysis Plan.

Thrombectomy alone versus intravenous Tenecteplase plus thrombectomy in patients with large vessel occlusion ischemic stroke: a comparison from SWIFT DIRECT and EXTEND-IA TNK Part 1 & 2 (SWIFT EXTEND)

Statistical Analysis Plan – 20/03/2024

1.) Inclusion / Exclusion Criteria based on target trial emulation:

a. Inclusion:

- Patient enrolled in SWIFT-DIRECT and received mechanical thrombectomy (MT) alone
- Patient enrolled in EXTEND-IA TNK Part 1 & 2 and received TNK 0.25 mg/kg before MT or TNK 0.40 mg/kg before MT
- Direct admission to a thrombectomy center with intention to treat with MT
- Age ≥ 18 years
- Neurological deficit with a National Institutes of Health Stroke Scale score (NIHSS) ≥ 5 points
- Pre-stroke independence (modified Rankin Scale (mRS) 0-2)
- Occlusion in the anterior circulation: intracranial internal carotid artery (ICA) and/ or the M1 segment, of the middle cerebral artery (MCA)
- Eligible for intravenous thrombolysis (IVT) within 4.5 hours
- Randomization within 4.5 hours

b. Exclusion:

- Life expectancy < 1 year
- Renal failure with creatinine > 3.0 mg/dl
- Occlusion in multiple vascular territories

2.) Aims and Hypothesis

The proposed project, named SWIFT EXTEND, is a causal inference study on observational data of SWIFT DIRECT and EXTEND-IA TNK Part 1 & 2 based on target trial emulation. We hypothesize that the exposure TNK 0++-0.25mg/kg or 0.40mg/kg within 4.5 hours after stroke onset before thrombectomy in patients with large vessel occlusion (LVO) acute ischemic stroke (AIS) will result in increased rates of functional independence (defined as mRS 0-2) compared to receiving MT alone.

Primary Aim:

- Assess whether TNK (0.25mg/kg or 0.40mg/kg) before MT results in higher rates of functional independence (mRS 0-2) at 90 days compared to MT alone.

Secondary Aims:

- To assess whether TNK (0.25mg/kg or 0.40mg/kg) before MT results in improved functional outcome (as assed by an ordinal shift analysis of the mRS at 90 days) compared to MT alone.
- To assess whether TNK (0.25mg/kg or 0.40mg/kg) before MT results in higher rates of freedom of disability (mRS 0-1) at 90 days compared to MT alone.
- To assess whether (0.25mg/kg or 0.40mg/kg) before MT changes safety outcomes compared to MT alone.
- To assess whether TNK (0.25mg/kg or 0.40mg/kg) before MT results in increased rates of early reperfusion prior to thrombectomy (on initial angiography runs or repeat CT perfusion/angiography) compared to MT alone.
- To analyze treatment interaction for the primary outcome in patients with onset to expected IVT time within or after 140 minutes.
- To analyze treatment interaction for the primary outcome over the whole range of onset to expected IVT time.
- Subgroup analysis to investigate whether MT within 30 minutes after administration of TNK (0.25mg/kg or 0.40mg/kg) results in higher odds for the primary outcome.
- Subgroup analysis to investigate whether TNK (0.25mg/kg) before MT results in higher odds for the primary outcome compared to MT alone.

Primary Endpoint:

Functional independence defined as a mRS score 0-2 at 90 days.

Secondary Endpoints:

- Functional outcome across the full ordinal mRS scale (merging categories 5 and 6) at 90 days
- Freedom of disability defined as mRS 0-1 at 90 days
- Early reperfusion prior to thrombectomy (defined as cross-sectional expanded Thrombolysis in Cerebral Infarction scale (eTICI) score of 2b – 3). In cross-sectional eTICI grading, the target downstream territory is defined based on findings on qualifying cross-sectional computed tomography angiography (CTA) or magnetic resonance angiography (MRA) and the extent of reperfusion is defined based on the first run of subsequent digital subtraction angiography.

Safety Outcomes:

- Intracranial hemorrhage at 24 ± 6 hours post randomization
- Symptomatic intracranial hemorrhage (sICH): any parenchymal hematoma type 1 (PH1), parenchymal hematoma type 2 (PH2), remote intracranial hemorrhage (RIH), subarachnoid hemorrhage (SAH), or intraventricular hemorrhage (IVH) associated with a ≥4 point worsening on the NIHSS within 24 hours

3.) Analysis

Causal inference study based on observational data from SWIFT DIRECT and EXTEND-IA TNK Part 1 & 2.

The dichotomous outcome functional independence (defined as an mRS of 0-2) at 90 days will be used as primary outcome as it was the primary outcome of one of the trials included in the analysis (SWIFT DIRECT). Additionally, functional outcome over the full ordinal mRS scale will be analyzed as a secondary outcome.

We will compare patients from SWIFT DIRECT with patients from EXTEND-IA TNK Part 1 & 2: SWIFT DIRECT will provide patients who received MT alone and EXTEND-IA TNK Part 1 & 2 will provide patients who received TNK 0.25mg/kg or 0.40mg/kg before MT. Only patients with direct admission to a stroke center will be included. We have identified potential confounders with the use of directed acyclic graphs (DAGs) (1,2) (**Figure 1**). Depending on the percentage of missing data on potential confounders and outcomes we will exclude patients from the analysis (in case of very low percentage of missing data) or use multiple imputation. Subsequently, we will estimate the average causal treatment effect of treatment allocation (direct MT versus Bridging with TNK) on primary and secondary outcomes by using standardization to population via

a) Inverse probability of treatment weighting (IPTW) including previously identified covariates (i.e. modelling the treatment) and

b) G-Computation approach (i.e. modelling the outcome)

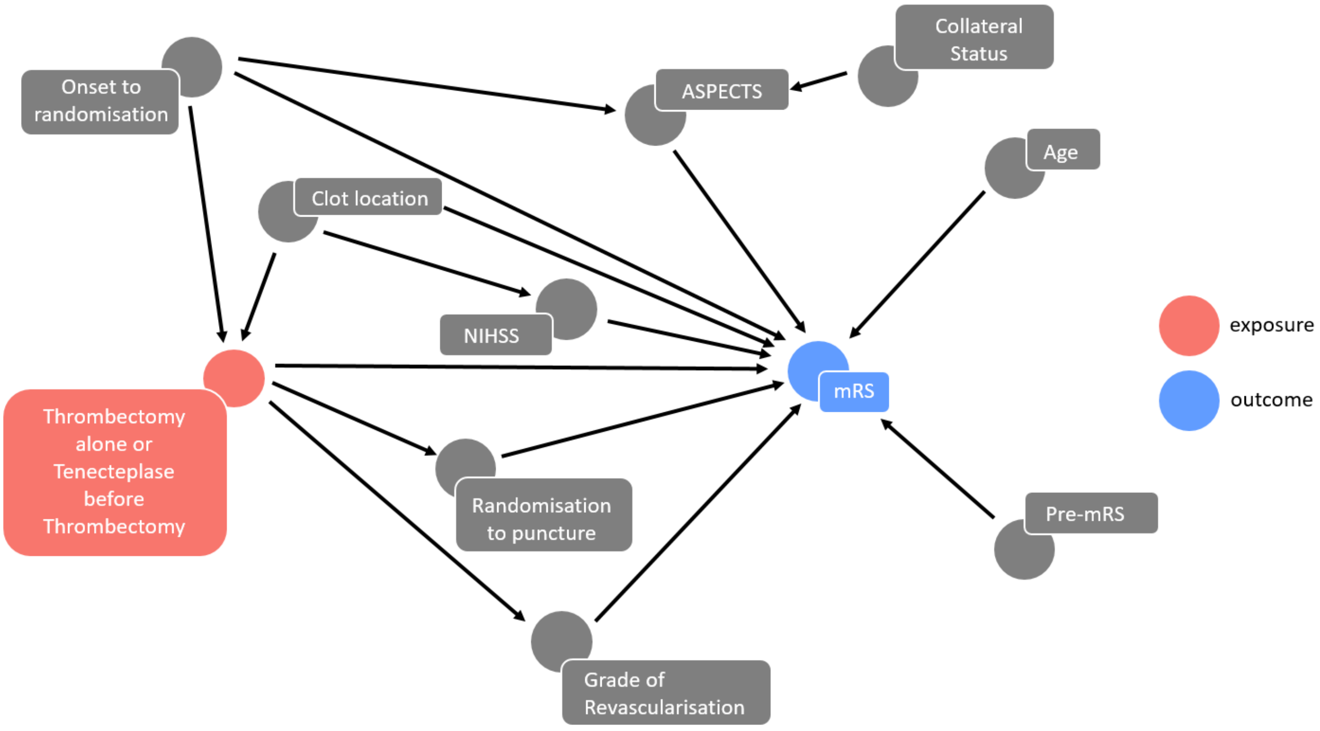

Figure 1: Directed acyclic graphs (DAGs). EVT = Endovascular Therapy. TNK = Tenecteplase. ASPECTS = Alberta Stroke Program Early CT Score. mRS = modified Rankin Scale. NIHSS = National Institutes of Health Stroke Scale.

In accordance with the DAGs, the statistical models for primary and secondary outcomes will be adjusted for age, stroke severity at onset (as measured by the NIHSS), occlusion location, time from onset to groin puncture and baseline Alberta stroke program early CT score (ASPECTS).

Participants who non-randomly violate positivity assumptions on the included covariates will be excluded. This way, the results will only be generalizable to the population that is based on the sample after such participants are excluded.

As TNK 0.40 mg/kg resulted in worse functional outcome and increased rates of intracranial hemorrhage in the NOR-TEST 2A trial (3) we will perform a subgroup analysis for the primary outcome only including patients treated with TNK 0.25mg/kg before MT and patients who received direct MT. To investigate a previously described time dependent effect on functional outcome of bridging thrombolysis with alteplase in comparison to direct MT (4) we will perform a treatment effect interaction analysis for the primary outcome using onset to expected IVT time as a dichotomous variable (within or after 140 minutes) as well as a continuous variable. Onset to expected IVT time will be calculated by adding the mean time from randomization to IVT of the respective trial to the time from onset to randomization of each patient as done in previous research (4).

Additionally, the primary outcome will be investigated in a further sensitivity analysis containing only patients who received MT within 30 minutes after thrombolysis with TNK (0.25mg/kg or 0.40 mg/kg).

Secondary endpoints will be analyzed within the same model used for the primary outcome. If the proportional odds assumption is not violated the functional outcome across the full ordinal mRS scale (merging categories 5 and 6) will be analyzed by IPTW and G-Computation. If proportional odds assumptions are violated, the oridnal analysis will revert to the use of probabilistic index model where G-Computation approach is feasible, but IPTW is not implemented.

4.) Limitations

The SWIFT-DIRECT and the EXTEND-IA TNK Part 1 & 2 trials were neither designed, nor powered to analyze outcomes in AIS patients receiving TNK and thrombectomy versus thrombectomy alone. In addition, the primary outcome of functional independence at 3 months was only the primary outcome in SWIFT direct whereas the primary outcome of EXTEND IA TNK Part 1&2 was reperfusion before thrombectomy. Indirect comparisons of populations from two different trials might also result in bias by both observed and unobserved cross-trial differences. Last, depending on the number of average baseline characteristics and the crosstrial baseline differences the effective sample size will be reduced.

5.) References

1. Digitale JC, Martin JN, Glymour MM. Tutorial on directed acyclic graphs. J Clin Epidemiol. 022;142:264-267.

2. VanderWeele TJ. Principles of confounder selection. Eur J Epidemiol. 2019;34(3):211-219.

3. Kvistad CE, Næss H, Helleberg BH, et al. Tenecteplase versus alteplase for the management of acute ischaemic stroke in Norway (NOR-TEST 2, part A): a phase 3, randomised, open-label, blinded endpoint, non-inferiority trial. Lancet Neurol. 2022;21(6):511-519.

4. Kaesmacher J, Cavalcante F, Kappelhof M, et al. Time to Treatment With Intravenous Thrombolysis Before Thrombectomy and Functional Outcomes in Acute Ischemic Stroke: A Meta-Analysis. JAMA. Published online February 7, 2024.

eMethods 3. Rationale and methods for subgroup "Time from tenecteplase to thrombectomy"

Prior studies have varied in whether shorter or longer time from thrombolytic to thrombectomy (dwell time) influences the benefit of bridging thrombolytic.[1,2] Longer dwell time is associated with greater pre-thrombectomy reperfusion but some studies suggested greater benefit when the alteplase infusion was still running during thrombectomy.[3,4] We therefore analysed the primary and secondary outcomes in subgroups defined by the time from tenecteplase to arterial puncture for thrombectomy (as a dichotomous variable within or after 30 minutes) in patients receiving bridging tenecteplase before thrombectomy. Adjusted OR or adjusted common OR and SRDs with 95% CIs were estimated via IPTW for the primary and secondary outcomes.

References

1. Zhu F, Gauberti M, Marnat G, et al. Time from I.V. Thrombolysis to Thrombectomy and Outcome in Acute Ischemic Stroke. Ann Neurol. Mar 2021;89(3):511-519.
2. Albers GW, Jumaa M, Purdon B, et al. Tenecteplase for Stroke at 4.5 to 24 Hours with Perfusion-Imaging Selection. N Engl J Med. Feb 22 2024;390(8):701-711.
3. Burian E, Sepp D, Lehm M, et al. Start, Stop, Continue? The Benefit of Overlapping Intravenous Thrombolysis and Mechanical Thrombectomy : A Matched Case-control Analysis from the German Stroke Registry. Clin Neuroradiol. Mar 2023;33(1):187-197.
4. Yogendrakumar V, Beharry J, Churilov L, et al. Tenecteplase Improves Reperfusion across Time in Large Vessel Stroke. Ann Neurol. Mar 2023;93(3):489-499.

List of investigators

SWIFT DIRECT:

Urs Fischer[1,2], Johannes Kaesmacher[3], Daniel Strbian[4], Omer Eker[5], Christoph Cognard[6], Patricia S Plattner[7], Lukas Bütikofer[8], Pasquale Mordasini[3], Sandro Deppeler[7], Vitor M Pereira[9], Jean François Albucher[10], Jean Darcourt[6], Romain Bourcier[11], Guillon Benoit[12], Chrysanthi Papagiannaki[13], Ozlem Ozkul-Wermester[14], Gerli Sibolt[4], Marjaana Tiainen[4], Benjamin Gory[15], Sébastien Richard[16], Jan Liman[17], Marielle Sophie Ernst[18], Marion Boulanger[19], Charlotte Barbier[20], Laura Mechtouff[21], Liqun Zhang[22], Gaultier Marnat[23], Igor Sibon[24], Omid Nikoubashman[25], Arno Reich[26], Arturo Consoli[27], Bertrand Lapergue[27], Marc Ribo[28], Alejandro Tomasello[29], Suzana Saleme[30], Francisco Macian[31], Solène Moulin[32], Paolo Pagano[33], Guillaume Saliou[34], Emmanuel Carrera[35], Kevin Janot[36], María Hernández-Pérez[37], Raoul Pop[38], Lucie Della Schiava[39], Andreas R Luft[40,41], Michel Piotin[42], Jean Christophe Gentric[43], Aleksandra Pikula[44], Waltraud Pfeilschifter[45], Marcel Arnold[2], Adnan H Siddiqui[46], Michael T Froehler[47], Anthony J Furlan[49], René Chapot[50], Martin Wiesmann[25], Paolo Machi[51], Hans-Christoph Diener[52], Zsolt Kulcsar[53], Leo H Bonati[1], Claudio L Bassetti[2], Mikael Mazighi[54], David S Liebeskind[55], Jeffrey L Saver[55], Jan Gralla[3]

EXTEND IA TNK:

Bruce CV Campbell[56], Peter J Mitchell, Leonid Churilov[56], Nawaf Yassi[56], Timothy J Kleinig[59], Richard J. Dowling, Bernard Yan[56], Steven J Bush, Vincent Thijs[57], Rebecca Scroop[61], Marion Simpson[60], Mark Brooks[62], Hamed Asadi[57,62,63], Teddy Y Wu[64], Darshan G Shah[65], Tissa Wijeratne[66], Henry Zhao[56], Fana Alemseged[56], Felix Ng[56], Peter Bailey[67], Henry Rice[68], Laetitia de Villiers[68], Helen M Dewey[69], Philip MC Choi[69], Helen Brown[65], Kendal Redmond[70], David Leggett[70], John N Fink[64], Wayne Collecutt[71], Thomas Kraemer[72], Martin Krause[73], Dennis Cordato[74], Deborah Field[75], Henry Ma[76], Bill O'Brien[77], Benjamin Clissold[78], Ferdinand Miteff[79], Anna Clissold[80], Geoffrey C Cloud[81], Leslie E Bolitho[82], Luke Bonavia[83], Arup Bhattacharya[84], Alistair Wright[85], Abul Mamun[86], Fintan O'Rourke[87], John Worthington[88], Andrew A Wong[89], Christopher R Levi[90], Christopher F Bladin[57,69,91], Gagan Sharma[56], Patricia M Desmond[58], Mark W Parsons[56], Geoffrey A Donnan[56], Stephen M Davis[56], Timothy Ang[88], Edrich Rodrigues[56], Patrick Salvaris[92], Carlos Garcia-Esperon[93], Claire Muller[65], Alan Coulthard[65], Ken Mitchell[70], John Clouston[70], Kate Mahady[70], Thanh G Phan[76], Winston Chong[94], Ronil V Chandra[95], Lee-Anne Slater[95], Timothy J Harrington[96], Kenneth C Faulder[97], Brendan S Steinfort[97]

Affiliations

1. Department of Neurology and Stroke Center, University Hospital Basel and University of Basel, Basel, Switzerland
2. Department of Neurology, University Hospital Bern, University of Bern, Bern, Switzerland
3. University Institute of Diagnostic and Interventional Neuroradiology, University Hospital Bern, Inselspital, University of Bern, Bern, Switzerland
4. Department of Neurology, Helsinki University Hospital, and University of Helsinki, Helsinki, Finland
5. Department of Neuroradiology, Hospices Civils de Lyon, Lyon, France
6. Department of Diagnostic and Therapeutic Neuroradiology, Centre Hospitalier Universitaire de Toulouse, Toulouse, France
7. Neuro Clinical Trial Unit, Department of Neurology, Inselspital, Bern University Hospital, University of Bern, Bern, Switzerland
8. CTU Bern, University of Bern, Bern, Switzerland
9. Division of Neurosurgery, Department of Surgery and Medical Imaging, St Michael's Hospital, University of Toronto, Toronto, ON, Canada
10. Department of Neurology, Centre Hospitalier Universitaire de Toulouse, Toulouse, France
11. Department of Diagnostic and Interventional Neuroradiology, Centre Hospitalier Universitaire de Nantes, Nantes Université, Nantes, France
12. Department of Neurology, Centre Hospitalier Universitaire de Nantes, Nantes Université, Nantes, France
13. Department of Radiology, CHU Rouen, Rouen, France
14. Department of Neurology, CHU Rouen, Rouen, France
15. Department of Diagnostic and Therapeutic Neuroradiology, INSERM U1254, CHRU-Nancy, Université de Lorraine, Nancy, France
16. Department of Neurology, Stroke Unit, INSERM U1116, CHRU-Nancy, Université de Lorraine, Nancy, France
17. Department of Neurology, University Medical Center Goettingen, Goettingen, Germany
18. Department of Neuroradiology, University Medical Center Goettingen, Goettingen, Germany
19. Department of Neurology, CHU Caen Normandie, University Caen Normandie, INSERM U1237, Caen, France
20. Department of Neuroradiology, CHU Caen Normandie, University Caen Normandie, INSERM U1237, Caen, France
21. Department of Vascular Neurology, Hospices Civils de Lyon, Lyon, France
22. Department of Neurology, St George's University Hospital NHS Foundation Trust, London, UK
23. Department of Interventional and Diagnostic Neuroradiology, CHU Bordeaux, University of Bordeaux, Bordeaux, France
24. Stroke Unit, CHU Bordeaux, University of Bordeaux, Bordeaux, France

25. Department of Neuroradiology, University Hospital RWTH Aachen, Aachen, Germany
26. Department of Neurology, University Hospital RWTH Aachen, Aachen, Germany
27. Department of Stroke and Diagnostic and Interventional Neuroradiology, Foch Hospital, Suresnes, France
28. Stroke Unit, Department of Neurology, Hospital Vall d'Heborn, Barcelona, Spain
29. Interventional Neuroradiology, Department of Radiology, Hospital Vall d'Heborn, Barcelona, Spain
30. Department of Neuroradiology, University Hospital of Limoges, Limoges, France
31. Department of Neurology, University Hospital of Limoges, Limoges, France
32. Department of Neurology, CHU Reims, Reims, France
33. Department of Neuroradiology, CHU Reims, Reims, France
34. Service of Interventional and Diagnostic Radiology, Centre Hospitalier Universitaire Vaudois and University of Lausanne, Lausanne, Switzerland
35. Department of Neurology, Hôpitaux Universitaires de Genève, Geneva, Switzerland
36. Department of Diagnostic and Interventional Neuroradiology, Tours University Hospital, Tours, France
37. Stroke Unit, Department of Neurosciences, University Hospital Germans Trias i Pujol, Barcelona, Spain
38. Department of Interventional Neuroradiology, Strasbourg University Hospitals, Strasbourg, France
39. Department of Neurology, Lille University Hospital, Lille, France
40. Department of Neurology, University Hospital of Zurich, Zurich, Switzerland
41. Cereneo, Center for Neurology and Rehabilitation, Vitznau, Switzerland
42. Department of Interventional Neuroradiology, Fondation Rothschild Hospital, Paris, France
43. Department of Neuroradiology, Brest University Hospital, Brest, France
44. Division of Neurology, Department of Medicine, Toronto Western Hospital, University of Toronto, Toronto, ON, Canada
45. Department of Neurology, University Hospital Frankfurt, Frankfurt, Germany
46. Department of Neurosurgery, Jacobs School of Medicine and Biomedical Sciences, University at Buffalo, New York, NY, USA
47. Vanderbilt Cerebrovascular Program, Vanderbilt University Medical Center, Nashville, TN, USA
48. School of Medicine, Case Western Reserve University, Cleveland, OH, USA
49. School of Medicine, Case Western Reserve University, Cleveland, OH, USA
50. Department of Intracranial Endovascular Therapy, Alfried Krupp Krankenhaus Essen, Essen, Germany
51. Department of Neuroradiology, Hôpitaux Universitaires de Genève, Geneva, Switzerland
52. Department of Neuroepidemiology, Institute for Medical Informatics, Biometry and Epidemiology IMIBE), Essen, Germany
53. Department of Neuroradiology, University Hospital of Zurich, Zurich, Switzerland
54. Department of Neurology, Lariboisière Hospital, University of Paris, FHU NeuroVasc, INSERM 1148, Paris, France
55. Department of Neurology and Comprehensive Stroke Center, David Geffen School of Medicine, University of California, Los Angeles, CA, USA
56. Department of Medicine and Neurology, Melbourne Brain Centre at the Royal Melbourne Hospital, University of Melbourne, Parkville, Victoria, Australia
57. The Florey Institute of Neuroscience and Mental Health, University of Melbourne, Parkville, Australia
58. Department of Radiology, the Royal Melbourne Hospital, University of Melbourne, Parkville, Victoria, Australia
59. Department of Neurology, Royal Adelaide Hospital, Adelaide, South Australia, Australia
60. Department of Neurology, Austin Hospital, Austin Health, Heidelberg, Victoria, Australia
61. Department of Radiology, Royal Adelaide Hospital, Adelaide, South Australia, Australia
62. Department of Radiology, Austin Hospital, Austin Health, Heidelberg, Victoria, Australia
63. School of Medicine, Faculty of Health, Deakin University, Victoria, Australia
64. Department of Neurology, Christchurch Hospital, Christchurch, New Zealand
65. Department of Neurology, Princess Alexandra Hospital, Brisbane, Queensland, Australia
66. Melbourne Medical School, Department of Medicine and Neurology, The University of Melbourne and Western Health, Sunshine Hospital, St Albans Victoria, Australia
67. Department of Neurology, Gold Coast University Hospital, Southport, Queensland, Australia
68. Department of Radiology, Gold Coast University Hospital, Southport, Queensland, Australia
69. Eastern Health and Eastern Health Clinical School, Department of Neurosciences, Monash University, Clayton, Victoria, Australia
70. Department of Radiology, Princess Alexandra Hospital, Brisbane, Queensland, Australia
71. Department of Radiology, Christchurch Hospital, Christchurch, New Zealand
72. Department of Medicine, Ballarat Base Hospital, Ballarat, Victoria, Australia
73. Department of Neurology, Royal North Shore Hospital and Kolling Institute, University of Sydney, St Leonards, New South Wales, Australia
74. Department of Neurology, Liverpool Hospital, Liverpool, New South Wales, Australia
75. Department of Neurology, Lyell McEwin Hospital, Adelaide, South Australia, Australia
76. School of Clinical Sciences, Department of Medicine, Monash University, Clayton, Victoria, Australia
77. Department of Neurology, Gosford Hospital, Gosford, New South Wales, Australia
78. Department of Neurology, University Hospital Geelong, Deakin University, Geelong, Victoria, Australia
79. Department of Neurology, Priority Research Centre for Brain and Mental Health Research, John Hunter Hospital, University of Newcastle, Newcastle, New South Wales, Australia
80. Department of Medicine, Southwest Healthcare, Warrnambool, Victoria, Australia
81. Department of Neurology, Alfred Hospital, Prahran, Victoria, Australia
82. Department of Medicine, Northeast Health, Wangaratta, Victoria, Australia
83. Department of Medicine, Albury Base Hospital, Albury, New South Wales, Australia
84. Department of Medicine, Goulburn Valley Health, Shepparton, Victoria, Australia

85. Department of Medicine, Latrobe Regional Health, Traralgon, Victoria, Australia
86. Department of Medicine, Campbelltown Hospital, Campbelltown, New South Wales, Australia
87. Department of Aged Care and Rehabilitation, Bankstown-Lidcombe Hospital, Bankstown, New South Wales, Australia
88. Department of Neurology, Royal Prince Alfred Hospital, Camperdown, New South Wales, Australia
89. Department of Neurology, Royal Brisbane and Women's Hospital and the University of Queensland, Brisbane, Queensland, Australia
90. Maridulu budyari gumal, The Sydney Partnership for Health Education Research & Enterprise (SPHERE), University of New South Wales, Sydney, Australia
91. Victorian Stroke Telemedicine service, Ambulance Victoria, Melbourne, Victoria, Australia
92. Department of Medicine, St John of God Midland Public and Private Hospitals, Perth, Western Australia
93. Hunter New England Local Health District, New Lambton Heights, NSW, Australia; Faculty of Medicine, University of Newcastle, Newcastle, NSW, Australia
94. Interventional Neuroradiology Unit, Monash Imaging, Monash Health, Clayton, Victoria, Australia; Faculty of Medicine, Nursing and Health Sciences, Monash University, Clayton, Victoria, Australia
95. Monash Imaging, Monash Health, Clayton, Melbourne, Australia; Department of Radiology, School of Clinical Sciences at Monash Health, Monash University, Melbourne, Victoria, Australia
96. Department of Neurosurgery, Australian School of Advanced Medicine, Macquarie University, Sydney, New South Wales, Australia; Department of Radiology, Royal North Shore Hospital, St Leonards, New South Wales, Australia
97. Department of Radiology, Royal North Shore Hospital, St. Leonards, Australia
